# A Novel Electrochemical Process for Desulfurization in the CaO-SiO_2_-Al_2_O_3_ System

**DOI:** 10.3390/ma13112478

**Published:** 2020-05-29

**Authors:** Sang Hoon Lee, Dong Joon Min

**Affiliations:** Department of Materials Science and Engineering, Yonsei University, 134 Shinchon-dong, Seodaemun-gu, Seoul 120-749, Korea; slag@yonsei.ac.kr

**Keywords:** electric potential, electro-desulfurization, oxidative desulfurization, sulfur evaporation

## Abstract

The effect of electric potential on the sulfide capacity of the CaO-SiO_2_-Al_2_O_3_ system was evaluated by applying voltages in the range of −1.5 to 1.5 V at 1823 K in a C/CO gas equilibrium. When the cathodic potential (−1.5 to 0 V) was applied, it was confirmed that the sulfur partition ratio increased based on the electrochemical reaction of sulfur (S + 2e^−^ = S^2−^). However, the reversibility of the electrochemical resulfurization reaction (S^2−^ = S + 2e^−^) in slag was not established in the reverse (anodic) potential region (0–1.5 V), yet the sulfur partition ratio increased. In particular, sulfur evaporation was observed in the anodic potential region. Therefore, in the present study, potential anodic electro-desulfurization mechanisms based on sulfur evaporation are proposed. To verify these mechanisms, sulfur evaporation is discussed in detail as a function of the thermodynamic stability of sulfur in the slag.

## 1. Introduction

The increasing importance of desulfurization during pyrometallurgy processes, which is attributed to the degradation of raw materials and increased demand for highly functional steel, makes the investigation of slag desulfurization an important endeavor.

Since Fincham and Richardson proposed a typical sulfur-refining mechanism at the slag/metal interface of CaO-SiO_2_-Al_2_O_3_ [1], sulfur has been known to be present in slag as stabilized sulfide or sulfate ions (depending on the oxygen partial pressure). Therefore, sulfur could be removed from the slag by transforming it into sulfides and sulfates through reduction and oxidation reactions, and controlling the oxygen partial pressure as follows:

(1) $$\left[S\right]+\left(O^{2-}\right)=\left(S^{2-}\right)+\frac{1}{2}O_{2}\;\left(p_{O_{2}}<10^{-5}\mathrm{atm}\right)$$

(2) $$\left[S\right]+\frac{3}{2}O_{2}+O^{2-}=\left({\mathrm{SO}}_{4}^{2-}\right)\;(p_{O_{2}}>10^{-3}\mathrm{atm})$$

Because the typical partial pressure of oxygen in pyrometallurgy is known to be stable in the sulfide region, studies have been mainly conducted on the reduction refining mechanism. For electrochemical applications, using Equation (1), the following electro-reduction desulfurization mechanism is proposed:

(3) $$\left[S\right]+2e^{-}=\left(S^{2-}\right)$$

Several kinetic studies have been conducted to enhance the chemical slag-refining capacity by accelerating the reaction of Equation (3) through the application of a direct electrical current [2,3,4,5,6,7,8,9]. Recently, it was confirmed that desulfurization can also be enhanced through voltage application at an electrochemical equilibrium. In other words, the electrochemical reduction of sulfur at the slag/metal interface based on Equation (3) is well established. Similarly, in the case of oxygen, the following electrochemical reaction is well known at the slag/metal interface:

(4) $$\left[O\right]+2e^{-}=\left(O^{2-}\right)$$

Additionally, the reversibility of the process by reversing the electric potential has been confirmed [10].

However, in the case of Equation (3), the electrochemical behavior of sulfur when reverse (anodic) potential is applied is still unclear. Using Equation (3), some researchers have reported that resulfurization occurs when a reverse (anodic) current is applied [3,4]. However, the results are in a quasi-equilibrium state and some studies on electro-slag remelting (ESR) have reported improved desulfurization with reverse current application. 

Thus, there is a lack of consensus on the electrochemical reversibility of sulfur when a reverse current is applied; furthermore, research on the application of a reverse voltage is limited.

Therefore, this study investigated the effect of a reverse (anodic) voltage on the behavior of sulfur and suggested possible desulfurization mechanisms based on the results.

## 2. Materials and Methods

The composition of the slag used in this study is shown in Table 1. The experimental materials and techniques for electrochemical refining used in this experiment are described in detail in our previous study [11]. After experiments, the sulfur content of the metal and slag was analyzed using an elemental analyzer (LECO CS-844). The silicon content of the metal was measured by inductively coupled plasma optical emission spectrometry (ICP-OES, Agilent 735). Sulfur evaporation was checked by performing inductively coupled plasma optical emission spectrometry (ICP-OES, Agilent 735) on the sulfur absorbed in the hydrogen peroxide solution of the outlet gas.

## 3. Results and Discussion

### 3.1. Cathodic Reaction of Sulfur

**Figure 1:**
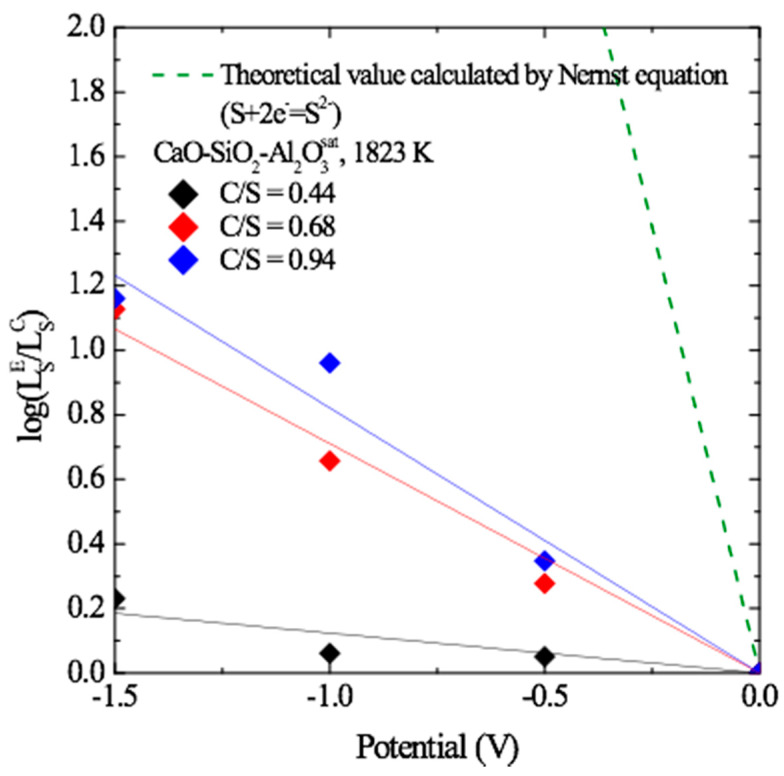
Sulfur partition ratio as a function of the cathodic potential in each of the three CaO-SiO_2_-Al_2_O_3_ (CSA) melts at 1823 K.

As a preliminary test, we verified the validity of Equation (3) in the slag composition of the current study. In Figure 1, the original sulfur concentration in liquid Cu is 1000 ppm. When there was no applied external voltage, the sulfur content in liquid Cu decreased to 684 ppm, 618 ppm and 527 ppm after slag refining for CaO/SiO_2_ (C/S) = 0.44, 0.68 and 0.94, respectively. It can be seen that the sulfur partition ratio increases as the voltage increases in the negative direction by the cathodic electro-desulfurization reaction (Equation (3)). At C/S = 0.94, the minimum concentration of sulfur in the metal was 81.2 ppm corresponding to the external voltage of −1.5 V and the sulfur partition ratio increased about 14 times. Using the Nernst equation, the correlation between the applied voltage and sulfur partition ratio is expressed as:

(5) $$\log\left(\frac{L_{S}^{E}}{L_{S}^{C}}\right)≅\frac{2F}{2.3\mathrm{RT}}\times \Delta E$$

where ΔE is the cell potential; $L_{S}=\left(wt\%S\right)/\left[wt\%S\right]$ is the sulfur partition between the slag and the metal; $L_{S}^{E}$ and $L_{S}^{C}$ are the sulfur partition ratios due to the cathodic potential and chemical potential, respectively; R = 8.314 JK/mol; F = 96.486 C/mol; and T is the temperature in K.

According to Equation (5), the sulfur partition ratio increases with increasing cathodic potential. The experimental results and the theoretical results (green dashed line in Figure 1) showed a poor correlation; additionally, the behavior of the partition ratio changed with the basicity of the slag. The discrepancy between the theoretical value and experimental results might be attributed to an ohmic drop (IR drop), bulk electrolysis and the dependence on the electrical conductivity and electrochemical properties of the slag used as the electrolyte [11]. Therefore, as the basicity of the slag increased, the electrical conductivity increased, reducing the effect of the IR drop.

These results are very consistent with those obtained in our previous study [11]. Thus, the electro-desulfurization demonstrated in Equation (3) was verified in the slag composition of the current experiment.

### 3.2. Anodic Reaction of Sulfur

Based on the aforementioned results, assuming that Equation (3) is a reversible reaction, the resulfurization reaction demonstrated in Equation (6) (in which the sulfur produced by the reoxidation of sulfide ions in the slag is dissolved into the metal) can be expected.

(6) $$\left(S^{2-}\right)=\left[S\right]+2e^{-}\;(\mathrm{resulfurization})$$

**Figure 2:**
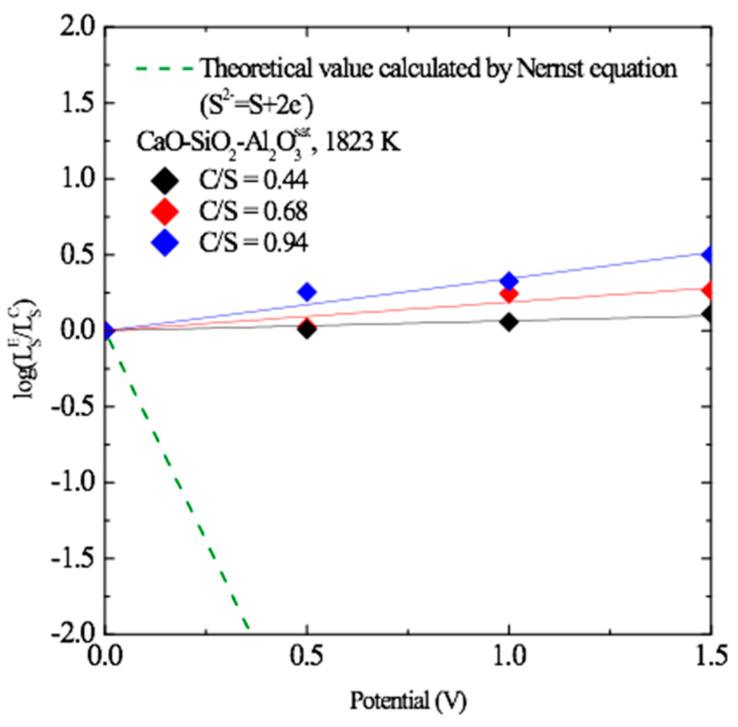
Sulfur partition ratio as a function of the anodic potential in the three CSA melts at 1823 K.

In this case, according to the theoretical correlation based on the Nernst equation, the sulfur partition ratio would be expected to decrease with voltage (green dashed line in Figure 2). However, after applying the reverse voltage, the sulfur partition ratio increased with voltage. At C/S = 0.94, the minimum concentration of sulfur in the metal was 170 ppm corresponding to the external voltage of 1.5 V and the sulfur partition ratio increased about three times. Therefore, Equation (6) was excluded.

**Figure 3:**
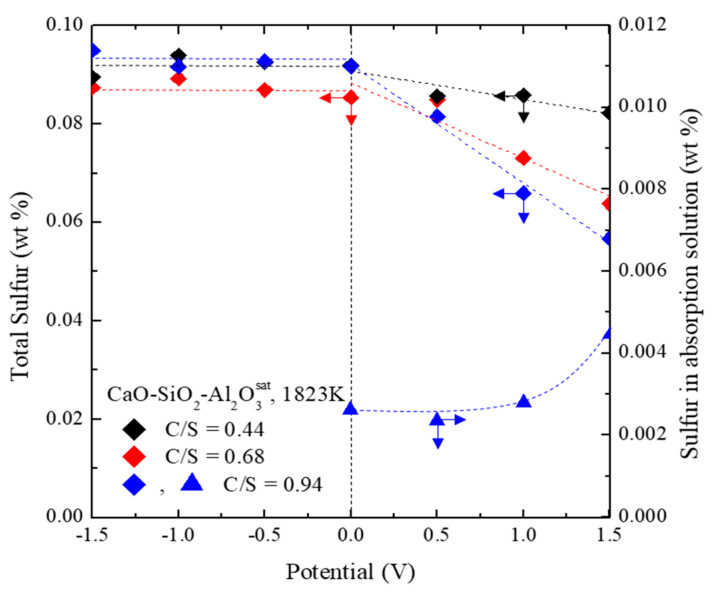
Residual sulfur concentration and sulfur concentration in the absorption solution after anodic electro-desulfurization as functions of the electrical potential.

After evaluating the amount of residual sulfur in the slag and metal after the experiment, the results revealed that the mass balance of sulfur was constant in the cathodic potential region but decreased in the anodic potential region (Figure 3). This is probably due to sulfur evaporation. In particular, in the case of C/S = 0.94, the maximum removal efficiency of residual sulfur due to sulfur evaporation was confirmed to be 38%. To confirm this, the gas from the outlet was passed through a 3% H_2_O_2_ aqueous solution for absorbing sulfur gas [12,13]. An increase in collected sulfur was observed as the applied anodic potential increased.

The sulfur evaporation in the slag is known to occur in the form of SO_2_ gas according to the following two reactions [14,15,16]:

(7) $$\left(S^{2-}\right)+\frac{3}{2}O_{2}={\mathrm{SO}}_{2}\left(g\right)+\left(O^{2-}\right)$$

(8) $$\left({\mathrm{SO}}_{4}^{2-}\right)={\mathrm{SO}}_{2}\left(g\right)+\frac{1}{2}O_{2}+\left(O^{2-}\right)$$

**Figure 4:**
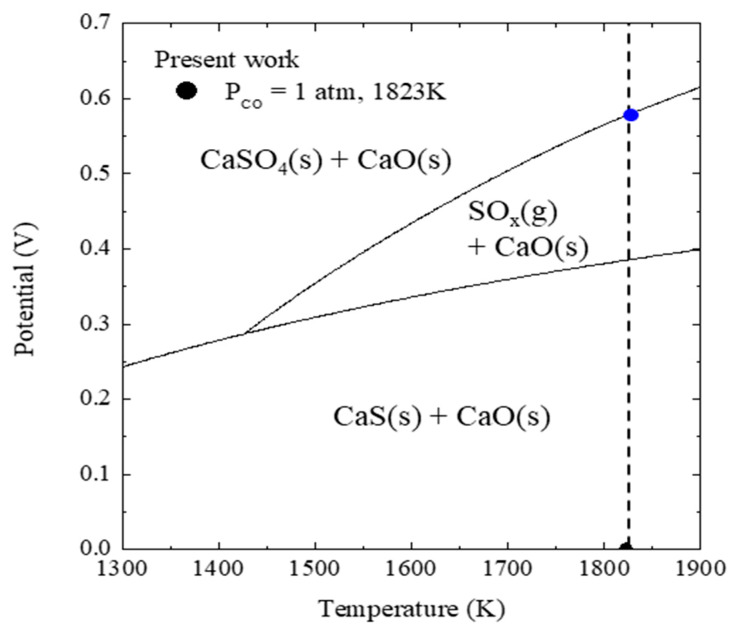
Thermodynamic stability of sulfur in the molten slag at 1823 K.

Based on Equations (7) and (8), and considering the case where sulfide and sulfate ions are stabilized by Ca^2+^ cations in the slag, the thermodynamic stable phase of sulfur is shown in Figure 4 [17]. 

Equation (7) indicates that the sulfide ions at an oxygen partial pressure $p_{O_{2}}=3\times 10^{-16}\mathrm{atm}$ (black dot in Figure 4) are oxidized to SO_2_ during the sulfate conversion process. Therefore, if an anodic potential is applied, the following anodic sulfur evaporation reaction (in which sulfide ions in the slag are oxidized to generate S_2_) can be predicted and S_2_ will be converted to SO_2_ according to the oxygen partial pressure:

(9) $$\left(S^{2-}\right)=\frac{1}{2}S_{2}\left(g\right)+2e^{-}\;\mathrm{anodic}\;\mathrm{sulfur}\;\mathrm{evaporation}$$

(10) $$\frac{1}{2}S_{2}\left(g\right)+O_{2}=SO_{2}$$

Then, because the sulfur concentration is locally reduced at the slag interface and an additional chemical driving force is provided, sulfur is expected to move from the metal into the slag to increase the sulfur partition ratio.

Equation (8) indicates that the sulfate ion in the slag was vaporized with SO_2_ in the process of being converted to sulfide as the oxygen partial pressure decreases. Therefore, if an anodic potential is applied, the following anodic desulfurization reaction in which sulfate ions are locally generated at the slag/metal interface can be predicted based on Equation (2):

(11) $$\left[S\right]+4O^{2-}=\left({\mathrm{SO}}_{4}^{2-}\right)+6e^{-}\;\mathrm{anodic}\;\mathrm{desulfurization}$$

Sulfate ions locally generated at the slag metal interface (blue dot in Figure 4) vaporize in the process of being converted to sulfide at an oxygen partial pressure $p_{O_{2}}=3\times 10^{-16}\mathrm{atm}$ (black dot in Figure 4) as they move to the bulk slag.

A similar electrochemical reaction mechanism exists for silicon:

(12) $$\left[\mathrm{Si}\right]+4O^{2-}=\left({\mathrm{SiO}}_{4}^{4-}\right)+4e^{-}$$

**Figure 5:**
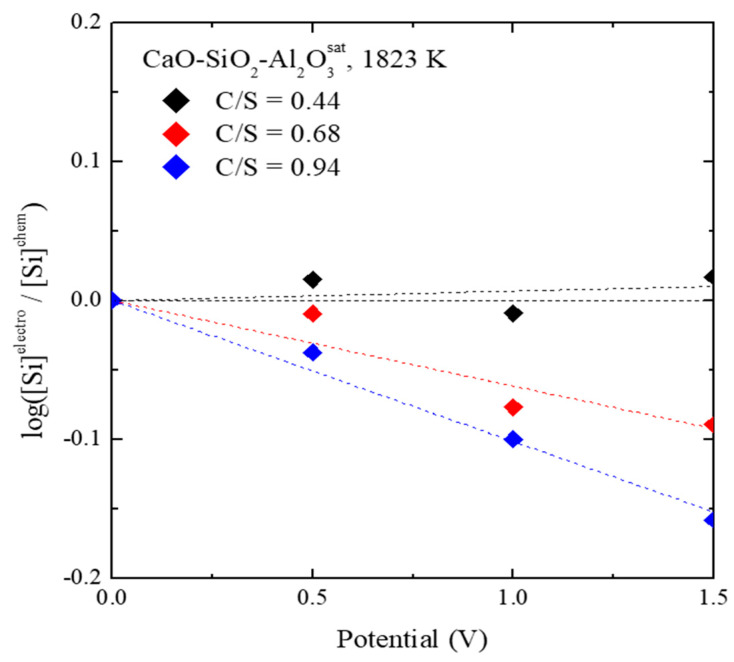
Silicon concentration in metal during anodic electro-desulfurization as a function of the anodic potential.

The Si concentration measured in the metal is shown in Figure 5 as a function of the applied anodic potential. It can be seen that the concentration of Si decreased when the anodic potential was applied. It is well known that Equation (12) works in the cathodic potential region as well [3,8,11,18]. This is because the stable phase of silicon ions in the slag is always determined only with ortho-silicate according to Pauling’s rule [19]. 

As a result, in the case of sulfur, the reversibility of the reaction was not satisfied. This is because sulfur evaporation occurs due to its instability. This increases during the conversion between the two equilibrium phases of sulfur in the slag when an anodic potential is applied.

**Figure 6:**
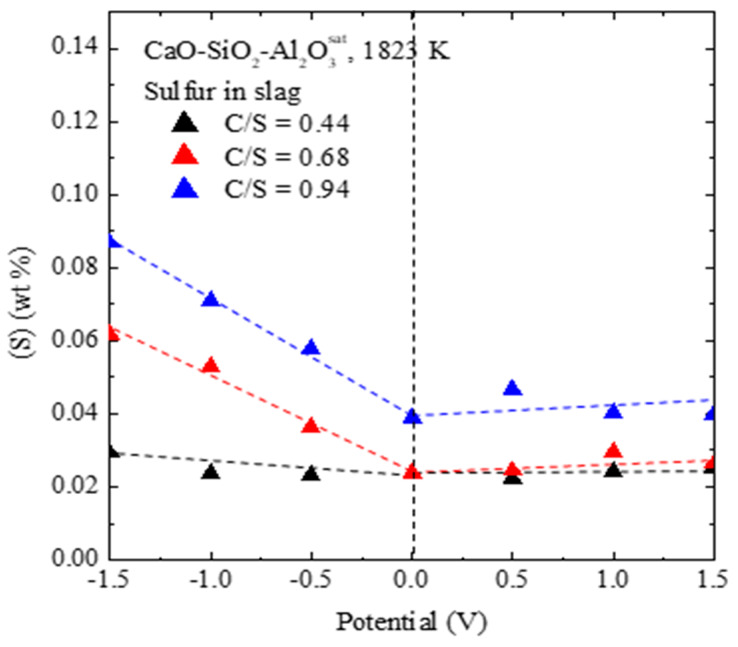
Effect of the electrical potential on residual sulfur concentration in the slag.

In addition, as shown in Figure 6, the anodic electro-desulfurization using the instability of sulfur in the slag results in a slight change its concentration. This means that the stability of sulfur in the slag is maintained after the anodic refining process, as compared to the previous cathodic refining process, which implies that slag recycling may be possible. Additionally, in the previous cathodic electro-desulfurization process, Si, Ca, and Al in the slag were reduced and contaminated with the molten metal [3,4,11], whereas our anodic electro-desulfurization process yielded high-purity metals, as shown in Figure 5.

## 4. Conclusions

**Figure 7:**
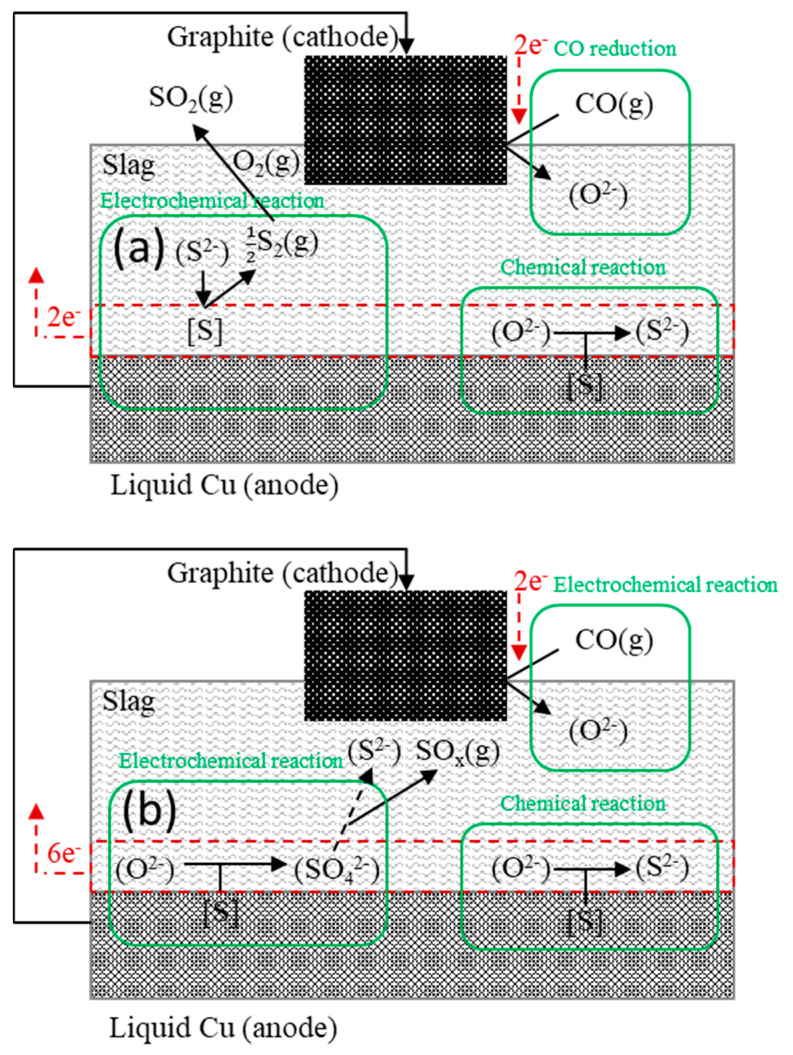
Proposed anodic electro-desulfurization mechanism in the CSA slag. (**a**) anodic sulfur evaporation (**b**) anodic desulfurization.

In this study, we proposed the mechanisms of anodic electro-desulfurization (Figure 7) which are described as follows:

(a) When an anodic potential is applied, the sulfide ions are oxidized to S through the reaction described by Equation (6). Because the produced S rapidly dimerizes and vaporizes in the form of S_2_ gas at high temperature, sulfide ions are locally scant at the slag/metal interface. Therefore, additional chemical potential is generated and sulfur is further removed from the metal.
(b) When an anodic potential is applied, sulfate ions are produced locally at the slag/metal interface according to Equation (8). The produced sulfate ions are converted to sulfide ions because of the low oxygen partial pressure ($p_{O_{2}}=3\times 10^{-16}\mathrm{atm}$) during their migration to the bulk slag. In this process, an intermediate product, SO_2_ gas, is present and thus sulfur evaporation occurs.

Based on the aforementioned mechanisms, this study confirms the improvement of the sulfur partition ratio when an anodic potential is applied. Currently, a study is being conducted to quantitatively evaluate the electrical effect of the anodic potential and to confirm sulfur evaporation directly. Thus, the present study is expected to provide a basis for further work on novel anodic electro-desulfurization.

## Figures and Tables

**Table 1:** Composition of CaO-SiO_2_-Al_2_O_3_ (CSA) melts.

	CaO (wt%)	SiO_2_ (wt%)	Al_2_O_3_ (wt%)	Basicity of Slag CaO/SiO_2_ (C/S) Ratio (wt%)	Initial Concentration of Sulfur in Metal (wt%)
CSA 1	17.6	40	42.4	0.44	0.1
CSA 2	22	32.5	45.5	0.68	0.1
CSA 3	24.8	26.4	48.8	0.94	0.1

## References

[1] Richardson F.D., Fincham C.J.B. (1954). Sulphur in silicate and aluminate slags. J. Iron Steel Inst..

[2] Ohtani M., Gokcen N.A. (1961). Physical Chemistry of Process Metallurgy.

[3] Ward R.G., Salmon K.A. (1963). Kinetics of sulphur transfer from iron to slag. 2. Effect of applied current. J. Iron Steel Inst..

[4] Kim D.H., Kim W., Kang Y.B. (2018). Electrochemical Transfer of S between Molten Steel and Molten Slag. Metall. Mater. Trans. B.

[5] Bills P.M., Littlewo R. (1965). Electrolytic Desulphurization of Molten Iron. J. Iron Steel Inst..

[6] Kato M., Hasegawa K., Nomura S., Inouye M. (1983). Transfer of oxygen and sulfur during direct current electroslag remelting. Trans. Iron Steel Inst. Jpn..

[7] Sen N., Ghosh M., Banerjee U.K., Mazumdar S., Ray H.S. (1999). Desulphurisation of high sulphur cast iron using an electrochemical technique. Scand. J. Metall..

[8] Kim D.H., Kim W., Kang Y.B. (2018). Electrochemical Desulfurization of Molten Steel with Molten Slag: Reaction Rate and Current Efficiency. J. Electrochem. Soc..

[9] El-Gammal T., Yostos B., Pakzad A. (1973). Indirect Electrolytic Desulphurization. Proc. Internat. Symp. Chem. Metall. Iron Steel.

[10] Kim W., Min D.J., Lee Y.S., Park J.H. (2009). Electrochemical method for controlling the interfacial oxygen in molten Fe with ZrO_2_ based solid electrolyte. ISIJ Int..

[11] Lee S.H., Min D.J. (2020). Effects of electrochemical potential on sulfur removal in the molten CaO–SiO_2_–Al_2_O_3_ system. Sep. Purif. Technol..

[12] Schoubye P., Christensen K.A., Nielsen M.T. (2010). Process for Removal of SO_2_ from Off-Gases by Reaction with H_2_O_2_. U.S. Patent.

[13] Zhou Y., Zhu X., Peng J., Liu Y., Zhang D., Zhang M. (2009). The effect of hydrogen peroxide solution on SO_2_ removal in the semidry flue gas desulfurization process. J. Hazard. Mater..

[14] Mori T., Moro-Oka A., Kokubo H. (1983). Gaseous Desulphurization from Metallurgical Slags. Tetsu-to-Hagané.

[15] Matsui A., Uchida Y.I., Kikuchi N., Miki Y. (2017). Effects of temperature and oxygen potential on removal of sulfur from desulfurization slag. ISIJ Int..

[16] Allertz C., Sichen D. (2015). Possibility of Sulfur Removal from Ladle Slag by Oxidation in the Temperature Range 1373–1673 K. J. Sustain. Metall..

[17] Turkdogan E.T. (1980). Physical Chemistry of High Temperature Technology.

[18] Sasaki H., Maeda M. (2015). Siliconizing of iron and molybdenum by electrochemical reduction of silicon in molten SiO_2_–Li_2_O–MgO. J. Alloys Compd..

[19] Pauling L. (1929). The principles determining the structure of complex ionic crystals. J. Am. Chem. Soc..

